# Polydopamine-Coated Manganese Complex/Graphene Nanocomposite for Enhanced Electrocatalytic Activity Towards Oxygen Reduction

**DOI:** 10.1038/srep31415

**Published:** 2016-08-16

**Authors:** Charlette M. Parnell, Bijay Chhetri, Andrew Brandt, Fumiya Watanabe, Zeid A. Nima, Thilak K. Mudalige, Alexandru S. Biris, Anindya Ghosh

**Affiliations:** 1 Center for Integrative Nanotechnology Sciences, University of Arkansas at Little Rock, 2801 South University Avenue, Little Rock, AR 72204, USA; 2 Department of Chemistry, University of Arkansas at Little Rock, 2801 South University Avenue, Little Rock, AR 72204, USA; 3 US Food and Drug Administration, Office of Regulatory Affairs, Arkansas Regional Laboratory, 3900 NCTR Road, Jefferson, Arkansas 72079, USA

## Abstract

Platinum electrodes are commonly used electrocatalysts for oxygen reduction reactions (ORR) in fuel cells. However, this material is not economical due to its high cost and scarcity. We prepared an Mn(III) catalyst supported on graphene and further coated with polydopamine, resulting in superior ORR activity compared to the uncoated PDA structures. During ORR, a peak potential at 0.433 V was recorded, which is a significant shift compared to the uncoated material’s −0.303 V (both versus SHE). All the materials reduced oxygen in a wide pH range via a four-electron pathway. Rotating disk electrode and rotating ring disk electrode studies of the polydopamine-coated material revealed ORR occurring via 4.14 and 4.00 electrons, respectively. A rate constant of 6.33 × 10^6^ mol^−1^s^−1^ was observed for the polydopamine-coated material–over 4.5 times greater than the uncoated nanocomposite and superior to those reported for similar carbon-supported metal catalysts. Simply integrating an inexpensive bioinspired polymer coating onto the Mn-graphene nanocomposite increased ORR performance significantly, with a peak potential shift of over +730 mV. This indicates that the material can reduce oxygen at a higher rate but with lower energy usage, revealing its excellent potential as an ORR electrocatalyst in fuel cells.

The electrochemical reduction of oxygen to water is an essential cathodic half reaction in proton exchange membrane fuel cells. Platinum-based catalysts are the most efficient materials for oxygen reduction reactions (ORR) and are currently used in commercial fuel cells^1^,^2^. However, this metal is expensive, scarce, and suffers from sluggish reaction kinetics and carbon monoxide poisoning, which diminishes its catalytic activity and longevity over time^3^,^4^. Alternatively, non-precious metal catalysts are an attractive replacement for platinum because they are cost effective, abundant, and electrochemically active in ORR^5^. Metal porphyrins and phthalocyanines complexes, such as iron (Fe) and cobalt (Co), have been widely used in ORR applications^6^,^7^. Unfortunately, these catalysts can suffer from demetallation, dissolution, and a short life cycle in the harsh pH environments of fuel cells, which limits their widespread use^8^,^9^. While pyrolysis is a traditional method for reducing metal degradation, it involves complex and inconsistent transformations of the chemical structure^10^. Therefore, in order to improve their activity and stability, carbon nanomaterials, such as graphene and multi-walled carbon nanotubes (MWCNTs), are being integrated as carbon supports for enhanced ORR performance.

Graphene and MWCNTs possess unique physical, morphological, electrical, and chemical properties, including a large surface area and electrical conductivity, which make them excellent candidates as active components in energy-related applications^11^,^12^,^13^,^14^. Metal complexes supported on carbonaceous nanomaterials, such as a Co-porphyrin supported on MWCNT^15^, a Mn tetrakis(4-hydroxyphenyl)porphyrin catalyst immobilized on poly(sodium-*p*-styrenesulfonate) modified reduced graphene oxide^16^, and an Fe-phthalocyanine anchored on single-walled carbon nanotubes^17^, have demonstrated excellent activities as ORR electrocatalysts. Previously, we have employed a Co(III) catalyst of an amidomacrocyclic ligand supported on graphene and MWCNTs for ORR^18^,^19^. The planar geometry of the complex, which contains empty coordination sites on the metal, along with multiple sources of π-electrons (i.e. benzene ring and lone pair electrons on oxygen and nitrogen atoms), allows it to easily attach to the carbon nanomaterial and quickly transfer electrons to the metal center for rapid reduction of oxygen to water^20^. The π-π interactions between the lone pair of electrons on the nitrogen and oxygen atoms in the ligand and the carbon nanomaterial may reduce their nucleophilicity, which may contribute to the overall activity and stability of the metal complex^21^. Although supporting metal complexes on carbon nanomaterials has proven to be efficacious in ORR, improvements in stability and activity can still be made in order to further reduce the electrochemical overpotential that is observed in these materials.

One widely-used method to develop superior ORR catalysts is the integration of heteroatom dopants such as nitrogen into the nanomaterial framework^22^,^23^,^24^,^25^. However, the nitrogen precursors that are used (i.e. ammonia, pyrrole, and pyridine) can be toxic^26^. Furthermore, the doping process can be time consuming and complicated, limiting their use^27^. Therefore, researchers are looking for alternative “green” nitrogen precursors with reduced toxicity and more favorable reaction conditions.

An example of a “green” nitrogen precursor material is polydopamine (PDA), which is non-toxic and biocompatible. PDA is an eco-friendly, bio-inspired mussel adhesive molecule that is generated by self-polymerization of dopamine, which forms a thin film on various substrates without any surface pretreatment^28^,^29^. It was previously used to develop ORR catalysts under metal and metal-free conditions^30^,^31^. The presence of nitrogen groups in PDA increases the electrical conductivity and, at the same time, enhances the electron affinity of the catalytic sites to facilitate adsorption of an oxygen molecule, which weakens the strong oxygen–oxygen double bond^21^. As a result, lower overpotential in ORR is observed. However, the use of PDA as a coating for metal complexes supported on a carbon nanomaterial to develop ORR catalysts has yet to be explored. Apart from increasing activity, PDA-coated structures of this nature can potentially benefit in overall stability, because PDA forms a protective barrier against harsh pH environments, which can help improve their widespread application in fuel cells.

Here, we present a nanocomposite that combines the usefulness and efficacy of metal complexes, carbon nanomaterials, and PDA to perform superior ORR. We have designed a Mn(III) catalyst of an amidomacrocyclic ligand supported on graphene structures and thoroughly characterized by a combination of analytical techniques. This nanocomposite was further coated with PDA at room temperature to enhance its reactivity and stability in ORR studies (Fig. 1). Electrochemical studies showed the PDA-Mn-graphene nanocomposite to have an ORR potential at 0.433 V (versus SHE), which is a dramatic shift from the Mn-graphene nanocomposite ORR potential at −0.303 V (versus SHE) and much closer to that of commercial platinum catalysts (367 mV difference)^32^. It is proposed that the improved ORR performance of the PDA-coated nanocomposite increased its electrical conductivity and interaction with oxygen due to a synergistic relationship between the PDA, graphene, and Mn(III) catalyst that allowed for easier and more rapid oxygen reduction. Hydrodynamic methods such as rotating disk electrode (RDE) and rotating ring disk electrode (RRDE) revealed that both nanocomposites reduce oxygen via a four-electron process with no detectable accumulation of hydrogen peroxide. Kinetically, the PDA-Mn-graphene nanocomposite reduced oxygen at 6.33 × 10^6^ mol^−1^s^−1^, which is, to our knowledge, the highest rate constant recorded among similar bioinspired synthetic catalysts employed for ORR.

## Results and Discussion

### Characterization of 2, Mn-graphene, and PDA-Mn-graphene nanocomposites

Infrared spectra of ligand **1** (Supplementary Figure S1) and Mn catalyst **2** are given in Supplementary Figure S2. The amide N-H stretching frequency appears at 3259 cm^−1^ in **1**. C-H stretching frequencies between 2967 and 2852 cm^−1^ in **1** are due to the sp^2^ and sp^3^ carbons in the aromatic ring and methyl groups. These stretching frequencies are slightly shifted to 3045 and 2852 cm^−1^ in **2**. bond carbonyl stretching frequencies were observed at 1668 and 1604 cm^−1^ in **1** and **2**, respectively. The C-N bond peak appeared at 1294 cm^−1^ in the ligand, which is slightly shifted to 1334 cm^−1^ in **2**. A strong peak at 525 cm^−1^ is seen in the catalyst spectrum but not present in the ligand, which could be due to the Mn-O stretching frequency that might originate between the interaction of **2** and a bound water molecule on the Mn metal center^33^. Electrospray ionization-mass spectrometry (ESI-MS) was also performed on **2** and showed a peak at 417 m/z (negative ion mode, Supplementary Figure S3), which is similar to the theoretical isotope distribution and calculated molecular weight of 417.04 g/mol (Supplementary Figure S3 insert).

**Figure 2:**
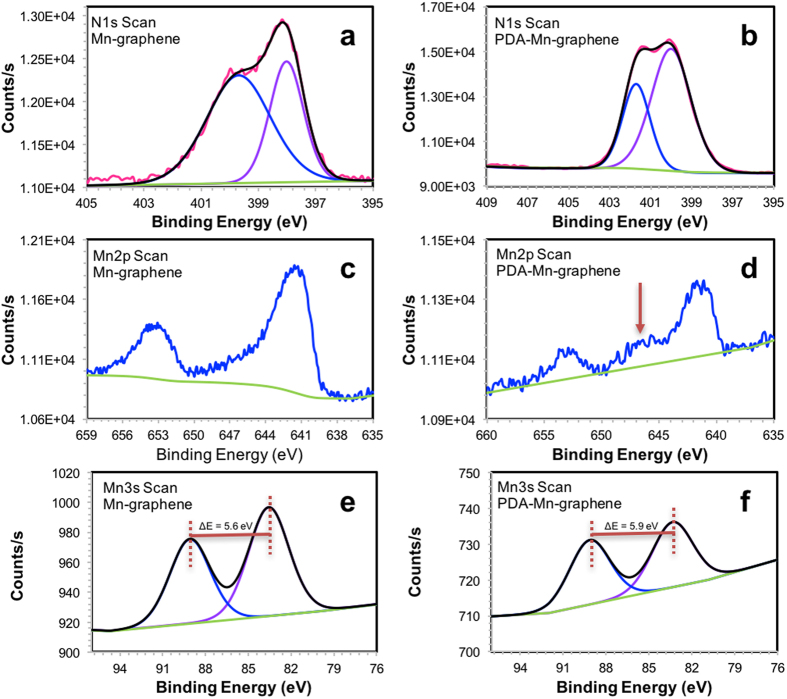
XPS narrow scans of Mn-graphene (**a**) N1s, (**c**) Mn2p and (**e**) Mn3s and PDA-Mn-graphene (**b**) N1s, (**d**) Mn2p and (**f**) Mn3s.

A series of X-ray photoelectron spectroscopy (XPS) analyses were run to determine the chemical states of some of the elements in the Mn-graphene nanocomposite and PDA-Mn-graphene nanocomposite (Supplementary Tables S1 and S2, respectively). Survey scans of the materials (Supplementary Figures S4a and S4b, respectively) were conducted followed by a narrow scans of specific elements. The binding energy of the Mn-graphene C1s narrow scan (Supplementary Figure S4c) was set to 284.0 eV for sp^2^ carbon bonds in the graphene nanomaterial^34^. The two peaks of the C1s scan of the PDA-Mn-graphene nanocomposite were set to 284.0 and 284.8 eV, corresponding to the sp^2^ and sp^3^ carbons, respectively (Supplementary Figure S4d). A narrow scan of N1s in the Mn-graphene nanocomposite gave two distinct binding energies (Fig. 2a). The first peak at 398.0 eV was due to the amide nitrogen groups in **2** and the peak at 399.7 eV was observed in the interaction between the nitrogen atoms in **2** and the carbon atoms in the graphene^35^,^36^. This peak shifted to 401.7 eV (Fig. 2b) in the PDA-Mn-graphene material, which has been attributed to the tautomeric species of polydopamine (5,6-dihydroxyindole and 5,6-indolequinone)^37^. O1s binding energies are indicative of the carbonyl oxygens found in **2** (Supplementary Figure S4e)^38^, with higher binding energies at 532.8 eV representative of single-bonded carbon-oxygen in PDA (Supplementary Figure S4f)^37^.

Mn2p and Mn3s narrow scans of the Mn-graphene nanocomposite were collected (Fig. 2c,d, respectively). The Mn2p scan showed two peaks at 642.1 and 653.7 eV, which gave a peak splitting and shape similar to that of Mn_2_O_3_^39^. Furthermore, the Mn3s narrow scan also showed two peaks at 84.24 and 89.96 eV. The peak splitting difference of 5.6 eV was similar to that seen in Mn(III) species, further confirming this oxidation state of **2** in the Mn-graphene nanocomposite^39^. Mn2p scan of the PDA-Mn-graphene nanocomposite gave a distinct satellite “hump” around 647 eV (indicated by red arrow), which is characteristic of MnO species (Fig. 2e). Also, the Mn3s scan gave a binding energy difference of 5.9 eV that matches well with Mn(II) species (Fig. 2f)^39^. These results indicate that during polymerization of dopamine to PDA and further interaction of PDA to **2**, the Mn center is reduced from +3 to +2 oxidation state.

**Figure 3:**
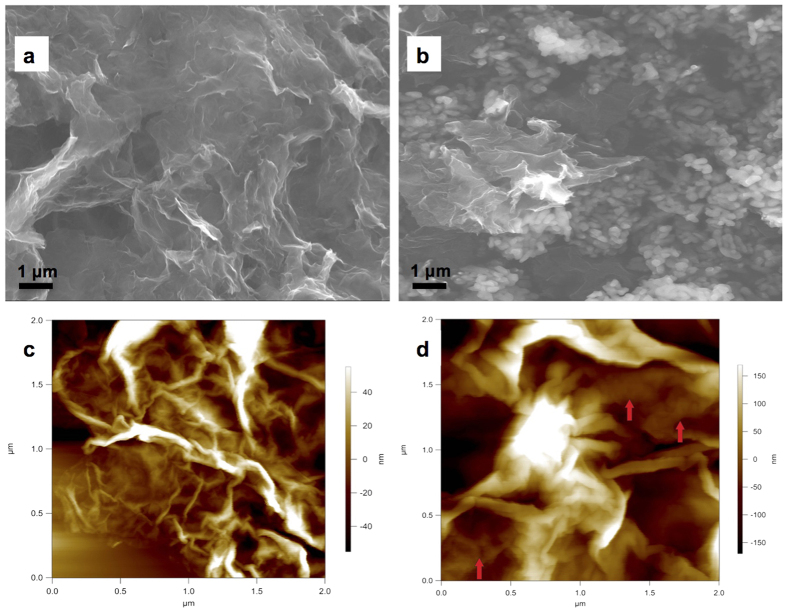
SEM images of (**a**) Mn-graphene and (**b**) PDA-Mn-graphene nanocomposites at x10,000 magnification; AFM images of (**c**) Mn-graphene and (**d**) PDA-Mn-graphene nanocomposites.

Scanning electron microscopy (SEM) image of the Mn-graphene nanocomposite (Fig. 3a) indicated a crinkled graphene material, which does not appear to show stacking of the graphene sheets. A similar appearance was seen in a Co nanocomposite on graphene previously utilized in ORR^18^. Transmission electron microscopy (TEM) image (Supplementary Figure S5a) showed a similar non-stacking pattern of the graphene sheets. Energy-dispersive X-ray spectroscopy (EDS) elemental mapping on the scanning transmission electron microscopy (STEM) image indicated the presence of Mn and nitrogen (from the amidomacrocylic ligand) atoms on the surface of the nanocomposite (Supplementary Figure S6a,c, respectively). The SEM of the PDA-Mn-graphene nanocomposite also indicated crinkled graphene sheets (Fig. 3b). In this image, the appearance of well-dispersed spherical structures can also be noted, which are attributed to the PDA coating; similar structures were also seen in other PDA-coated materials^40^,^41^. The TEM image (Supplementary Figure S5b) indicated a coating on the graphene sheets (green arrows), which contributes to the PDA coating on the Mn-graphene nanocomposite. STEM with EDS analysis revealed the presence of widely dispersed Mn atoms (Supplementary Figure S6b) and uniformly coated nitrogen atoms (Supplementary Figure S6d) on the surface of the PDA-Mn-graphene nanocomposite.

Atomic force microscopy (AFM) imaging of the Mn-graphene (Fig. 3c) and PDA-Mn-graphene nanocomposites (Fig. 3d) showed similar results to the SEM, including crinkled graphene sheets. Figure 3d showed spherical structures that were also seen in other PDA-coated materials^42^. In addition, we also observed that the PDA-Mn-graphene nanocomposite was softer than the graphene sheets, and the spherical shapes deformed or flattened when imaged with a higher set point (harder tapping force on sample).

Raman spectroscopy of the Mn-graphene and the PDA-Mn-graphene material were compared, and the spectra are given in Supplementary Figure S7. Both spectra give D and G bands at 1344 and 1579 cm^−1^ (Mn-graphene) and 1345 and 1570 cm^−1^ (PDA-coated material), which has been observed in graphene materials. The G band is indicative of the graphene carbons, and the D band indicates the defect present within the graphene structure. The slight shift in the two bands in PDA-Mn-graphene was due to the presence of PDA, an effect that has been reported earlier^43^. Moreover, the ratio between the graphene D and G bands intensity values (indicating disorder/defects) (I_D_/I_G_) changed slightly from 1.53 to 1.11 in the Mn-graphene and PDA-coated materials, respectively. In the lower range of the spectra, the presence of **2** is observed. Supplementary Figure S8 shows the Raman spectra between 200 and 600 cm^−1^. The Mn-N bond in **2** is observed at 410 cm^−1^ in the Mn-graphene nanocomposite and slightly shifted to 416 cm^−1^ in the PDA-coated material^44^.

### Electrochemical studies of Mn-graphene nanocomposite

**Figure 4:**
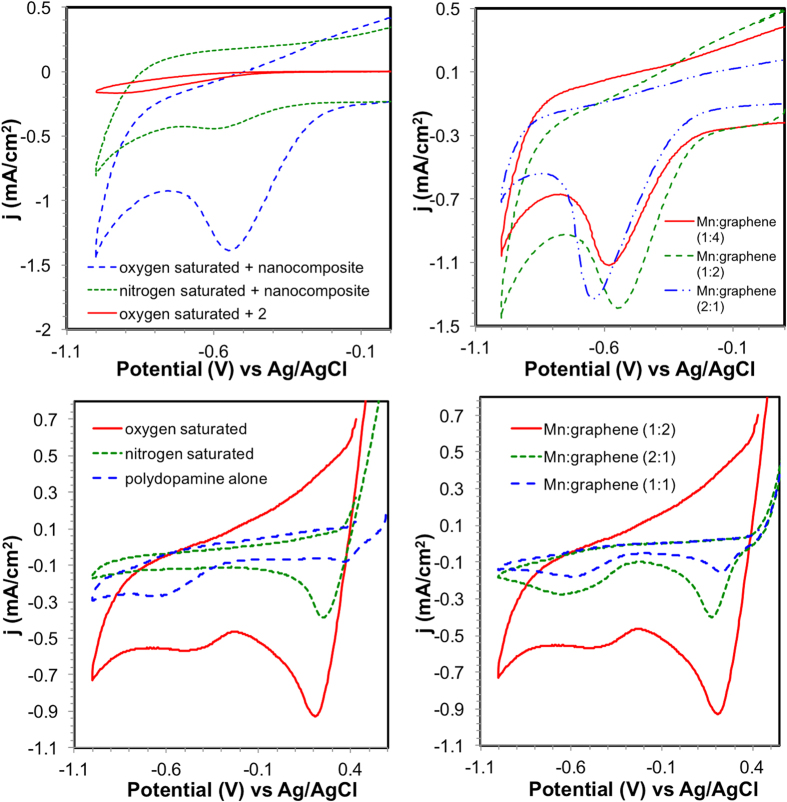
CV of (**a**) Mn-graphene nanocomposite in N_2_- and O_2_-saturated solutions at pH 2.0 and (**b**) the effect of Mn catalyst:graphene ratio at pH 2.0. Effect of (**c**) PDA coating on Mn-graphene nanocomposite in N_2_- and O_2_-saturated solutions at pH 2.0 and (**d**) changing Mn:graphene ratio in PDA-coated material.

The electrochemical activity of the nanocomposite to catalytically reduce oxygen was evaluated using cyclic voltammetry (CV). Initial evaluation of the Mn-graphene nanocomposite compared the efficacy of the material under an oxygen (O_2_)- and nitrogen (N_2_)-saturated pH 2.0 electrolyte solution. As is shown in Fig. 4a, the nanocomposite showed very little reduction of O_2_ and a low current density under anaerobic conditions. However, when the study was performed in an O_2_-saturated solution, the current density displayed a significant increase toward ORR. Additionally, the peak potential shifted positively about +60 mV.

Electrochemical studies of **2** were performed under similar conditions. As shown in Fig. 4a, the catalyst alone exhibited no significant activity towards ORR. The reduction peak at −0.653 V (versus SHE) is most likely due to the reduction of Mn metal from +3 to +2 oxidation state. Graphene was also tested in a similar manner and showed no significant ORR activity. However, when **2** was supported on graphene, a synergistic effect was created, giving the nanocomposite a high current density and affinity towards ORR activity. Its peak potential was observed at −0.303 V (versus SHE), with an approximate current density value of 1.37 mA/cm^2^.

Next, the effect of the type of nanomaterial was tested by comparing graphene- and MWCNT-supported electrocatalysts. As Supplementary Figure S9 shows, the graphene material exhibited a higher current density than MWCNT during ORR. Additionally, the peak potential shifted positively +163 mV, giving it lower ORR overpotential. During electrochemical studies, the graphene allowed more efficient transportation of electrons through the material to reach the catalyst active site, resulting in quick oxygen reduction and less overpotential^13^. It also showed higher current density. A similar observation was also shown for a previously published Co nanocomposite supported on graphene when compared to MWCNT^19^. Thus, we found that the graphene to be a better catalyst support material for ORR.

The ratio of **2** and graphene was compared to determine the optimal pairing of the two. As seen in Fig. 4b, when 1:2 ratio is casted, the current density gives the best performance. A 1:4 ratio diminished the amount of oxygen reduced, most likely due to decreased amount of Mn active sites relative to the graphene surface. When twice as much **2** was grafted onto graphene, the current density decreased slightly. However, the peak potential shifted negatively with a slightly higher overpotential than the 1:2 ratio sample. At this point, the graphene material was being saturated with **2**. The decreased oxygen reduction and higher overpotential resulted from the smaller amount of graphene reducing the ability to transfer electrons between the nanomaterial and **2**.

### Electrochemical studies of PDA-Mn-graphene nanocomposite

We then studied the effect of PDA on the Mn-graphene nanocomposite to observe any positive influences on peak potential and/or current density. Figure 4c shows the CV of the PDA-Mn-graphene material. There are several peaks to note. First, the small broad peak at −0.277 V (versus SHE) corresponds with the electrochemical redox reaction involving PDA. When tested alone, the same broad peak is also observed in PDA, confirming this phenomenon. Secondly, the peak at 0.433 V (versus SHE) is attributed to the reduction of oxygen. This peak is also observed in the N_2_-saturated solution with a much lower current density. In comparison to the Mn-graphene nanocomposite, the peak potential has a significant +730 mV shift. When compared to the PDA-coated graphene nanocomposite (no Mn catalyst), the current density of the PDA-Mn-graphene material was almost twice as large and, therefore, demonstrates the need for the Mn catalyst for higher ORR activity.

A possible explanation for the dramatic shift observed in the ORR potential lies within the interaction between PDA and graphene. The nitrogen atoms in PDA interacts with the sp^2^ carbon network in graphene, which creates defects in the adjacent sites. This, in turn, changes in the charge density, resistance in charge transfer, and hydrophilicity of the material to assist in ORR^45^. Moreover, the charge density change can affect the contact of the dissolved oxygen molecules on the PDA-Mn-graphene nanocomposite. The oxygen bond is weakened, allowing for easier ORR. Thus, when PDA interacts with graphene, the conjugated system is delocalized between the sp^2^ carbon framework in graphene and the lone pairs of electrons on the nitrogen in PDA to give better electrochemical transfer towards ORR with a lower overpotential.

Ratio studies were also performed with the PDA-Mn-graphene nanocomposite to observe any changes in the current density and/or peak potential (Fig. 4d). From the CV, the best ratio was achieved with 1:2, followed by 2:1 and 1:1. This trend was similarly seen in the Mn-graphene nanocomposite (Fig. 4b), with 1:2 ratio giving the greatest current density and peak potential for ORR.

### pH studies of nanocomposites

The Mn-graphene nanocomposite was further tested in a wide range of pH conditions. Supplementary Figures S10 and S11 show the voltammograms in pH 2.0 to 10.0 for the Mn-graphene and PDA-Mn-graphene materials, respectively. As the pH became more alkaline, a negative shift in peak potential was observed. This could be due to a different reaction mechanism of oxygen reduction at acidic versus alkaline conditions. Furthermore, it is proposed that a water molecule is attached to the Mn center in **2** (as noted in Supplementary Figure S2). In an alkaline pH, this water molecule can easily become deprotonated, which affects the affinity of oxygen during ORR. It is also noted that the PDA-Mn-graphene material gave similar current densities in acidic and alkaline pH (compared to Mn-graphene). Typically, amidomacrocyclic metal catalysts of this nature are very unstable in acidic pH^9^. However, the PDA and graphene coating have provided additional stability for the catalyst in acidic and alkaline media for enhanced ORR performance. In order to confirm the type of mechanism occurring in each media, further testing using linear sweep voltammetry was performed.

### RDE and RRDE studies

**Figure 5:**
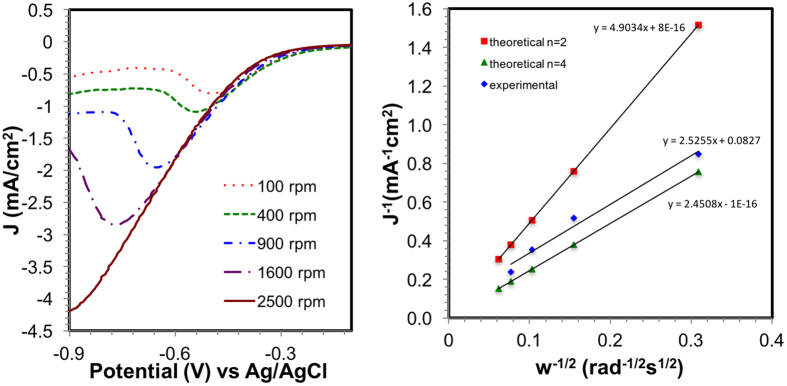
(**a**) RDE studies at pH 2.0 and (**b**) the corresponding Koutecky-Levich plot for the Mn-graphene nanocomposite.

RDE was conducted in both pH 2.0 and 10.0 at an increasing number of rotations. In pH 2.0, RDE (Fig. 5a) showed that the current density increased when the rotation rate increased. To determine the mechanism, the limiting currents from the RDE were used in Koutecky-Levich analysis to approximate the number of electrons involved in ORR. The plot shows a linear relationship between the limiting (J_lim_), Levich (J_Lev_) and kinetic currents (J_k_). Theoretical slopes where the number of electrons, n, equals 2 and 4 were also plotted for comparison. From the slope, the Levich equation was used to calculate the number of electrons in the electrochemical reaction.

Figure 5b shows the corresponding Koutecky-Levich plot at pH 2.0. The experimental slope closely matches that of a theoretical n = 4. The calculated number of electrons was 3.88 electrons, which indicated a four-electron pathway. When conducted in alkaline media at pH 10.0, the RDE showed similar voltammetry sweeps with increased current density at higher rotation rates (Supplementary Figure S12). The corresponding Koutecky-Levich plot revealed that the experimental slope matched well with theoretical n = 4 with a calculated number of electrons of 4.22. When compared to the PDA-Mn-graphene material at pH 10.0 (Supplementary Figure S13), the calculated number of electrons was 4.14.

The rate constant was determined for each experimental condition by the kinetic current using the equation J_k_ = 10^3^ nFkCΓ, where k is the rate constant and Γ is the concentration of the catalyst deposited on the electrode surface. In acidic media, the rate constant was 1.3 × 10^6 ^mol^−1^s^−1^, which is larger than that of a similar Co electrocatalyst^18^. When compared to other ORR electrocatalysts that have been developed, this rate constant is higher than that of a Co-MWCNT electrocatalyst (1.62 × 10^5 ^mol^−1^s^−1^)^19^ and a Co-graphene catalyst (3.85 × 10^5 ^mol^−1^s^−1^)^18^. In alkaline conditions, the rate constant decreased slightly to 2.50 × 10^5 ^mol^−1^s^−1^. The decrease resulted from the higher overpotential encountered at higher pH, which decreased the rate of ORR. The PDA-Mn-graphene nanocomposite gave a rate constant of 6.33 × 10^6 ^mol^−1^s^−1^, which is over twenty-four times higher than the non-PDA-coated nanocomposite at pH 10.0 and higher than that of a Co-porphyrin-MWCNT electrocatalyst (1.3 × 10^6 ^mol^−1^s^−1^)^15^, cofacial Co_2_-porphyrin (3.0 × 10^5 ^mol^−1^s^−1^)^46^, and a Co-monocorrole catalyst (5.7 × 10^5 ^mol^−1^s^−1^)^47^ previously used in ORR (Table 1). As mentioned earlier, the delocalization of charge, caused by integrating nitrogen in the carbon network, assisted the reduction of oxygen at a much faster rate. The changes in rate constants between the Mn-graphene and PDA-Mn-graphene nanocomposite revealed that doping with PDA greatly enhanced ORR activity. Table 1 compares the ORR activity of the Mn-graphene and PDA-Mn-graphene nanocomposites with similar ORR electrocatalysts. From this comparison, the PDA-Mn-graphene nanocomposite had a peak potential with a lower overpotential than other ORR electrocatalysts, which indicates lower energy usage of the material. We would also like to note that our rate constant is slightly lower than that of a biosynthetic hybrid electrocatalyst of cytochrome *c* oxidase^48^. To the best of our knowledge, it is notably the highest rate constant achieved during ORR compared to other synthetic catalysts.

We also conducted RRDE, which possesses a platinum ring surrounded by a graphite disk. The nanocomposite was deposited onto the disk, carefully avoiding the platinum ring. As the electrode rotates at a constant speed, the initial oxygen reduction occurs at the disk. Any formation of peroxide intermediate is diffused outward towards the platinum ring, where it is further reduced to water and any accumulation of peroxide will be observed by a ring current.

**Figure 6:**
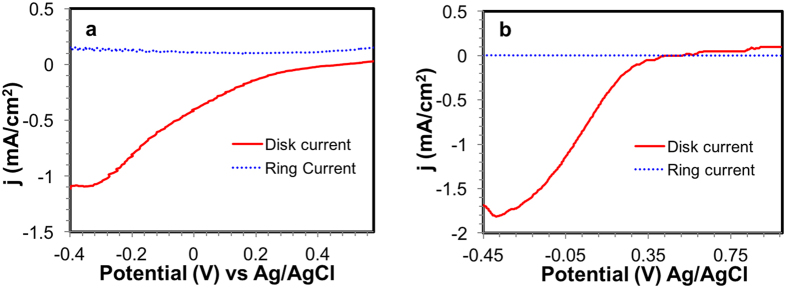
RRDE studies of (**a**) Mn-graphene and (**b**) PDA-Mn-graphene nanocomposites at pH 2.0.

The RRDE in pH 2.0 is plotted for the Mn-graphene (Fig. 6a) and the PDA-Mn-graphene material (Fig. 6b). Likewise, RRDE studies in alkaline conditions (pH 10.0) are given in Supplementary Figure S14a,b for the Mn-graphene and the PDA-Mn-graphene nanocomposite, respectively. The ring current did not increase appreciably in either sample, implying that no intermediate is accumulating in large amounts at the platinum ring and suggesting a one-step four-electron reduction process. The number of electrons calculated from RRDE was 3.99 for the Mn-graphene nanocomposite and 4.00 for the PDA-Mn-graphene nanocomposite at pH 2.0. In alkaline conditions, the number of electrons reflected a four-electron process for both materials with a value of 4.00 for both Mn-graphene and PDA-coated material.

### Proposed mechanism

**Figure 7:**
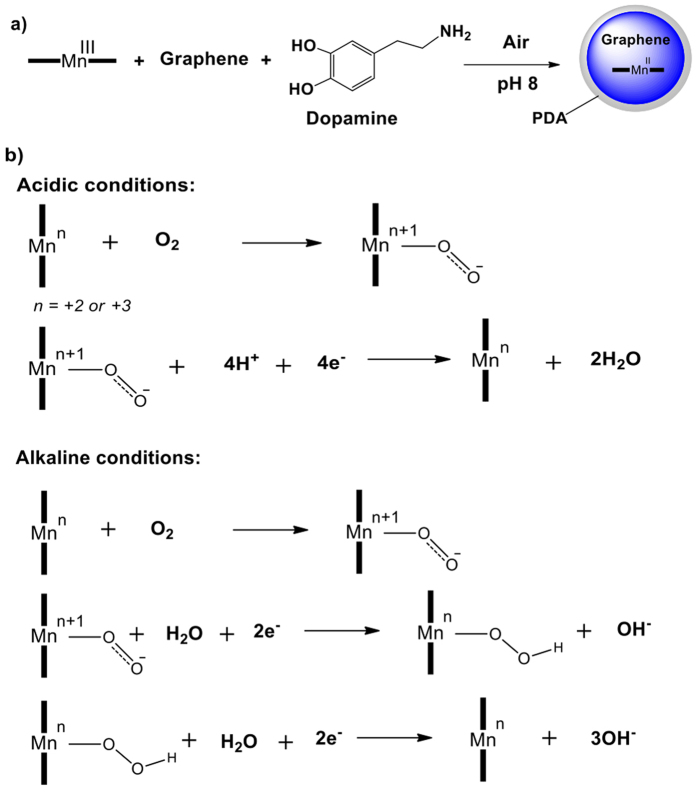
(**a**) Possible reduction pathway of Mn(III) to Mn(II) with PDA-coated material and (**b**) proposed mechanism of Mn-graphene or PDA-Mn-graphene nanocomposite in acidic and alkaline conditions.

The proposed general mechanism of the Mn-graphene nanocomposite in acidic and alkaline media is given below. From the XPS results, the Mn2p and Mn3s narrow scans showed the presence of a Mn(III) species in the Mn-graphene nanocomposite. It has been previously shown that PDA’s catechol groups can covalently graft with metal salts and even reduce the metal ions to their corresponding metallic form^49^,^50^,^51^. In this study, during the coating of PDA on the nanocomposite, reduction of the Mn(III) catalyst to Mn(II) was observed, as indicated by XPS analysis of the PDA coated material (Fig. 7a). Low-valent metal complexes, such as Mn(II) complexes, and oxygen-activating metallo-enzymes are more susceptible to reaction with oxygen than their high-valent counterparts^48^,^52^. Figure 7b gives the general proposed mechanism in acidic and alkaline conditions^53^. We represent the catalytic center as Mn(n), where n = +2 or +3 (in the case of the PDA-Mn-graphene nanocomposite or the Mn-graphene nanocomposite, respectively). We believe that in ORR, the Mn center is oxidized to a high-valent oxidation state from an “n” oxidation state during the initial interaction with oxygen. For example, in acidic media if Mn existed in an Mn(III) state, an oxygen molecule binding to the catalyst would generate an Mn(IV) intermediate. Further reduction of oxygen to water regenerates the Mn(III) oxidation state and completes the catalytic cycle. Alternatively, alkaline conditions proceed via a different intermediate due to the increased concentration of hydroxide ions (OH^−^) in higher pH. The Mn(III) interacts with oxygen and water in the presence of two electrons to yield a hydroperoxo intermediate. Next, the hydroperoxide species is rapidly reduced via a two-electron process to hydroxide and regenerates the Mn(III) catalyst.

### Stability studies

In order to show the effect of acidic media on the Mn catalyst’s stability, we conducted stability studies. The Mn-graphene modified electrode was left in pH 2.0 phosphate buffer solution for several days. Periodically, CV was performed to observe changes in the peak potential position and/or current density. Supplementary Figure S15 gives an overview of the stability studies while Supplementary Figure S16 shows a more detailed account. After the first 24 hours, the current density decreased slightly with a loss of 4.79% (Supplementary Figure S15). Between 24 hours and 96 hours, little to no change was observed in current density. As seen in Supplementary Figure S16, the current density remained fairly constant, while the peak potential shifted slightly. The inset shows the changes in current density over the first 24 hours. The small changes that occurred could be due to less dissolved oxygen in the solution.

Studies with the alkaline medium were also conducted using ultraviolet-visible spectroscopy (UV/Vis) to observe any changes in the absorbance of **2**. Using time dependence studies, UV/Vis was conducted for **2** and after the addition of the pH 10.0 buffer solution (Supplementary Figure S17). Scans were measured intermittently for a total of five minutes in order to observe the initial stability of **2** in alkaline media. At t = 0 (red line), a peak at 580 nm is associated with the dark yellow solution of **2**. This peak has been found in other Mn(III) complexes^54^ and has been shown to be attributed to the ligand-to-metal charge transfer that occurs between the amide nitrogen p-orbital and the d-orbital of the Mn(III) metal center^55^. When t = 0 and t = 5 scans were compared, no significant changes in this peak were observed. The inset is given for clarity to show no change in the absorbance. The small decrease in the peak absorbance of the Mn catalyst at t = 0 compared to t = 5 is attributed to the decreased concentration of the Mn catalyst when dissolved in the alkaline buffer.

## Conclusions

In this study, an Mn(III)-amidomacrocyclic complex was synthesized and used for the first time in ORR activity. The catalyst was supported on graphene and tested in a wide range of pH. CV of the nanocomposite showed increased current density in comparison to the Mn catalyst and graphene alone. This revealed that the synergy between the catalyst and graphene produces a nanocomposite capable of reducing oxygen more efficiently than the two individual components. When the nanocomposite was further coated with PDA, the multicomponent material revealed a significant increase in ORR peak potential from −0.303 V to 0.433 V (both versus SHE).

Varying the pH revealed increased current density in alkaline media accompanied with slightly higher overpotential. The PDA-coated nanocomposite also showed catalytic activity in a wide pH range, which is advantageous in fuel cell applications. Reaction kinetics from the hydrodynamic studies revealed a four-electron pathway with one of the highest rate constants—6.33 × 10^6 ^mol^−1^s^−1^—for quick reduction of oxygen. During the formation of the PDA coating, the Mn(III) complex was reduced to a Mn(II) complex. This oxidation state change, along with the possible interaction of PDA with graphene, furnished a nanocomposite with enhanced ORR activity. Furthermore, the RRDE ring current did not increase, which suggests that no hydroperoxide intermediate is involved in the ORR mechanism. To our knowledge, this is the first use of a PDA-Mn-graphene nanocomposite for ORR applications.

## Methods

### General

Chemicals used in this study were purchased from Sigma-Aldrich, USA, Acros Organics, USA, or VWR International and were used without further purification unless otherwise noted. Graphene was obtained from Angstron Materials (N002-PDR Graphene Powder, 97% purity) and used without any additional modification. MWCNTs were purchased from Bayer MaterialScience (Baytubes C150P RD SAM) and used as received. Nitrogen gas (ultrahigh purity) and oxygen gas (ultrahigh purity) were obtained from NLR Welding Supply Inc. Buffer solutions of different pH (2.0, 4.0, 7.0, 8.0, 9.0, 10.0, and 12.0) were prepared using published procedure^56^ and further diluted with deionized water. Infrared spectra were collected using a Thermo Scientific Nicolet 6700 FT-IR spectrometer. ESI-MS was collected using an Agilent 1100 series MSD Trap VL spectrometer. UV/Vis spectra were recorded using a Varian Cary 5000 UV–Vis–NIR spectrophotometer. Elemental analysis was obtained from Midwest Microlab LLC, Indianapolis, Indiana, USA. CV analyses were conducted using a Pine Instrument (Grove City, PA) bipotentiostat (Model AFCBP1) at 25 °C. SEM was carried out by using JEOL SEM (JSM 7000F). Raman analysis was done with a Raman spectrometer (Horiba Jobin Yvon LabRam HR800, Edison, New Jersey) under the excitation of a He-Ne laser (514 nm wavelength and 17 mW-at the sample surface) connected to a Peltier-cooled CCD camera. All the studies were done by using the 100X objective, which is attached to an Olympus BX-51 microscope. All spectra were acquired at room temperature by using the 600-line∕mm gratings and with an identical acquisition time. Before any studies, the Raman spectrometer was thoroughly calibrated using the Si-Si Raman peak located at 521 cm^−1^ Raman shift.

### Synthesis of ligand (1) and Mn(III) complex (2)

The amidomacrocyclic ligand **1** was synthesized as previously described (Supplementary Figure S1)^57^,^58^. Synthesis of the manganese complex was performed as follows. In a 100-mL Schlenk flask equipped with a magnetic stir bar, ligand **1** (300 mg, 0.67 mmol) was added and dissolved with dry THF (20 mL) under N_2_. The temperature was lowered to 0 °C by placing the flask in an ice bath. Once the solution was cooled, *n*-butyllithium (1.10 mL, 2.7 mmol, 2.5 M in hexanes) was added, followed by anhydrous manganese(II) chloride (87 mg, 0.67 mmol). The reaction was slowly heated to room temperature and allowed to stir overnight. The reaction yielded a yellowish-brown precipitate. The solution was exposed to air to allow the metal to oxidize from Mn(II) to Mn(III). The solvent was removed *via* rotary evaporator to yield a solid yellowish brown product and dried under vacuum. Yield: 75% (247.5 mg). λ_max_ = 408 nm, ε = 1.21 × 10^2 ^M^−1^cm^−1^. This catalyst was used in aqueous solutions for all the UV/Vis studies. The remaining studies used **2** with PPh_4_^+^ as the counter ion, which was synthesized following literature procedure^20^. The water-soluble Mn(III) catalyst **2** was dissolved in distilled water, from which the PPh_4_^+^ salt of the catalyst was precipitated using PPh_4_Cl. The precipitate was filtered and dried under vacuum. Anal. Calcd for [C_43_H_34_MnN_4_O_4_P]·H_2_O: C, 66.66; H, 4.68; N, 7.23. Found: C, 66.92; H, 4.50; N, 7.37%.

### Preparation of Mn-graphene nanocomposite

5 mg of **2** was dissolved in 5 mL of methanol. From this solution, 2 mL of the Mn catalyst was transferred to a new vial. 4 mg of the carbon nanomaterial (MWCNTs or graphene) was added to the solution and methanol was further added to give a 1 mg/mL suspension. The mixture was sonicated for 30 min. 40 μL of Nafion^®^ was added and the suspension was sonicated for an additional 30 min. Other ratios used in this study were prepared similarly by changing the amount of **2** and carbon nanomaterial.

### Preparation of PDA-Mn-graphene nanocomposite

The PDA-coated nanocomposites were prepared by following literature procedure with some alterations^59^. In a 100 mL round bottom flask, 5 mg of **2** and 10 mg of graphene were added and suspended in 50 mL NaOH solution (pH 8). This was sonicated for 15–30 min. 125 mg (0.659 mmol) dopamine was added to the suspension and vigorously stirred for 3 h. 40 μL of Nafion^®^ was added and the suspension was sonicated for an additional 30 min. Additional ratios of the PDA-coated nanocomposite were prepared similarly by changing the amount of **2** and graphene used.

### Electrochemical studies

CV was performed with a potential range of 0.6 V to −1.0 V (versus Ag/AgCl) at a scan rate of 100 mV/s, unless otherwise noted. CV studies were performed in a three-electrode system using a glassy carbon working electrode, Ag/AgCl reference electrode, and platinum counter electrode. The glassy carbon working electrode was then modified with the previously prepared Mn-graphene or PDA-Mn-graphene nanocomposite. A 10 μL aliquot of the nanocomposite was drop casted onto the electrode and dried at room temperature. Within the electrochemical cell, a phosphate buffer solution was added and purged with either oxygen or nitrogen gas for at least 1 h prior to electrochemical testing. The peak potential values are given with respect to SHE.

### X-ray photoelectron spectroscopy

XPS studies were carried out by using a Thermo Scientific Model K-Alpha XPS instrument using monochromatic Al Kα radiation (1486.7 eV) with the X-ray spot size 200 μm for each sample. The base pressure in the analysis chamber was typically 1 × 10^−9 ^mbar. Samples were then mounted to the sample holder using double-sided tape. All spectra were collected with the charge neutralization flood gun turned on. The typical pressure during the analysis with the flood gun on was 2 × 10^−7 ^mbar. The collected data were processed using the Thermo Scientific Advantage XPS software package. Spectral charge correction was performed using the main C1s peak due to hydrocarbon (C-C/C-H bonds) set to 284.8 eV. Mixed Gaussian-Lorentzian peak shapes as well as a Shirley/Smart type background subtraction were used for the peak analysis and fitting.

### Transmission Electron Microscopy

TEM observations of nanocomposite samples were performed by JEOL JEM 2100F equipped with a field emission gun at 80 kV acceleration voltage and an EDS (EDAX Corporation) used for elemental analysis. Each sample was dispersed into ethanol and a few drops were deposited and allowed to dry on a TEM holey carbon covered copper grid.

### Atomic force microscopy

Nanocomposite was suspended in methanol, and the resulting solutions were pipetted onto freshly cleaved mica (Ted Pella Inc., Redding, CA). Tapping mode AFM images were acquired using an Asylum Research MFP-3D AFM. Silicon probes (ACST-SS probe, Applied Nano Structures, Inc., Mountain View, CA) were used with force constants of 7–8 N/m, nominal resonant frequencies of 150 kHz, and average tip radios less than 6 nm.

## Additional Information

**How to cite this article**: Parnell, C. M. *et al*. Polydopamine-Coated Manganese Complex/Graphene Nanocomposite for Enhanced Electrocatalytic Activity Towards Oxygen Reduction. *Sci. Rep.* **6**, 31415; doi: 10.1038/srep31415 (2016).

## Supplementary Material

Supplementary Information

## Figures and Tables

**Figure 1:**
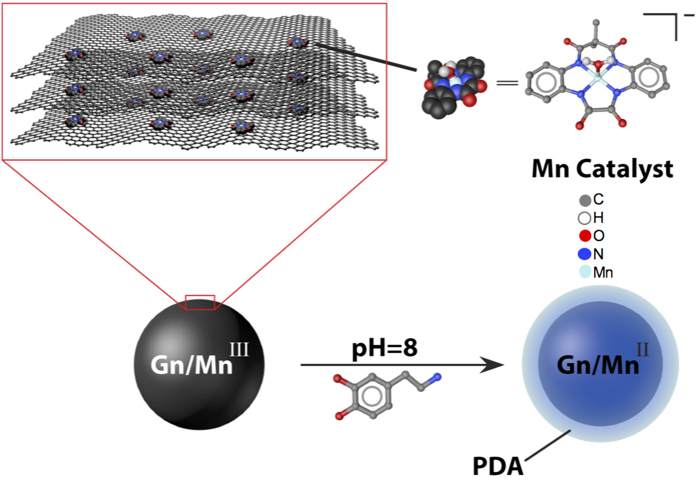
Preparation of polydopamine (PDA)-coated Mn-graphene (Gn/Mn) nanocomposite.

**Table 1:** Comparison study of recent ORR electrocatalysts.

ORR Catalyst	ORR Peak Potential (V) *vs.* SHE	Rate Constant (mol^−1^s^−1^)	Number of Electrons	Reference
Co-porphyrin-MWCNTs	0.447	1.8 × 10^6^	4.2	15
MnTHPP/PSS-rGO	−0.079	—	3.72	16
FePc-Py-CNT	0.915	—	4.05	17
Co-graphene	0.237	3.85 × 10^5^	4.04	18
Co-MWCNT	0.043	1.62 × 10^5^	3.95	19
3D-N-RGO/MnO	−0.153	—	3.03	23
NCF-Co	0.027	—	3.96	27
Co@Co_3_O_4_@PPD	−0.027	—	3.96	30
Co_2_(FTF4)	0.661	3.0 × 10^5^	4.0^*a*^	46
(Me_4_Ph_5_Cor)Co	0.621	5.7 × 10^5^	2.9	47
G65YCu_B_Mb	−0.263	1.98 × 10^7^	4.0^*a*^	48
Fe(III)/N/C HNSs-750	0.027	—	3.8	60
**Mn-graphene**	**−0.303**	**1.3 × 10**^**6**^	**3.88**	**This work**
**PDA-Mn-graphene**	**0.433**	**6.33 × 10**^**6**^	**4.14**	**This work**

*Co-porphyrin-MWCNTs: cobalt-porphyrin-multiwalled carbon nanotubes; MnTHPP/PSS-rGO: manganese 5,10,15,20-tetrakis(4-hydroxphenyl) porphyrin/poly(sodium-*p*-styrenesulfonate)-reduced graphene oxide; FePc-Py-CNT: iron phthalocyanine-pyridyl-carbon nanotubes; 3D-N-RGO/MnO: three-dimensional nitrogen-doped reduced graphene oxide/manganese monoxide; NCF-Co: nitrogen-doped carbon fibers on cobalt; Co@Co_3_O_4_@PPD: cobalt@cobalt oxide core@shell nanoparticles embedded in pyrolyzed polydopamine; Co_2_(FTF4): dicobalt(face-to-face) porphyrin; (Me_4_Ph_5_Cor)Co: 7,8,12,13-tetramethyl-2,3,10,17,18-pentaphenylcorrolato)-cobalt(III); G65YCu_B_Mb: G65 tyrosine mutant of distal Cu_B_ functionalized on myoglobin; Fe(III)/N/C HNSs-750: iron(II,II) oxide nanoparticles on nitrogen-doped hollow nanospheres annealed at 750 °C. ^*a*^Approximate number of electrons.
