# Supplementary material for: Polydopamine-Coated Manganese Complex/Graphene Nanocomposite for Enhanced Electrocatalytic Activity Towards Oxygen Reduction
Source: Sci Rep. 2016 Aug 16;6:31415. doi: 10.1038/srep31415 (PMC4985631; doi:10.1038/srep31415)
Supplement: Supplementary Information [file srep31415-s1.pdf]

Supplementary Information

Polydopamine-Coated Manganese Complex/Graphene Nanocomposite for Enhanced Electrocatalytic Activity Towards Oxygen Reduction

Charlette M. Parnell[1], Bijay Chhetri[2], Andrew Brandt[2], Fumiya Watanabe[1], Zeid A. Nima[1], Thilak K. Mudalige[3], Alexandru S. Biris[1], Anindya Ghosh[2*]

[1]*Center for Integrative Nanotechnology Sciences, University of Arkansas at Little Rock, 2801 South University Avenue, Little Rock, AR 72204, USA*

[2]*Department of Chemistry, University of Arkansas at Little Rock, 2801 South University Avenue, Little Rock, AR 72204, USA*

[3] *U.S. Food and Drug Administration, Office of Regulatory Affairs, Arkansas Regional Laboratory, 3900 NCTR Road, Jefferson, Arkansas 72079, USA*

*corresponding author

E-mail: axghosh@ualr.edu, Phone: 501 569 8827, Fax: 501 569 8838

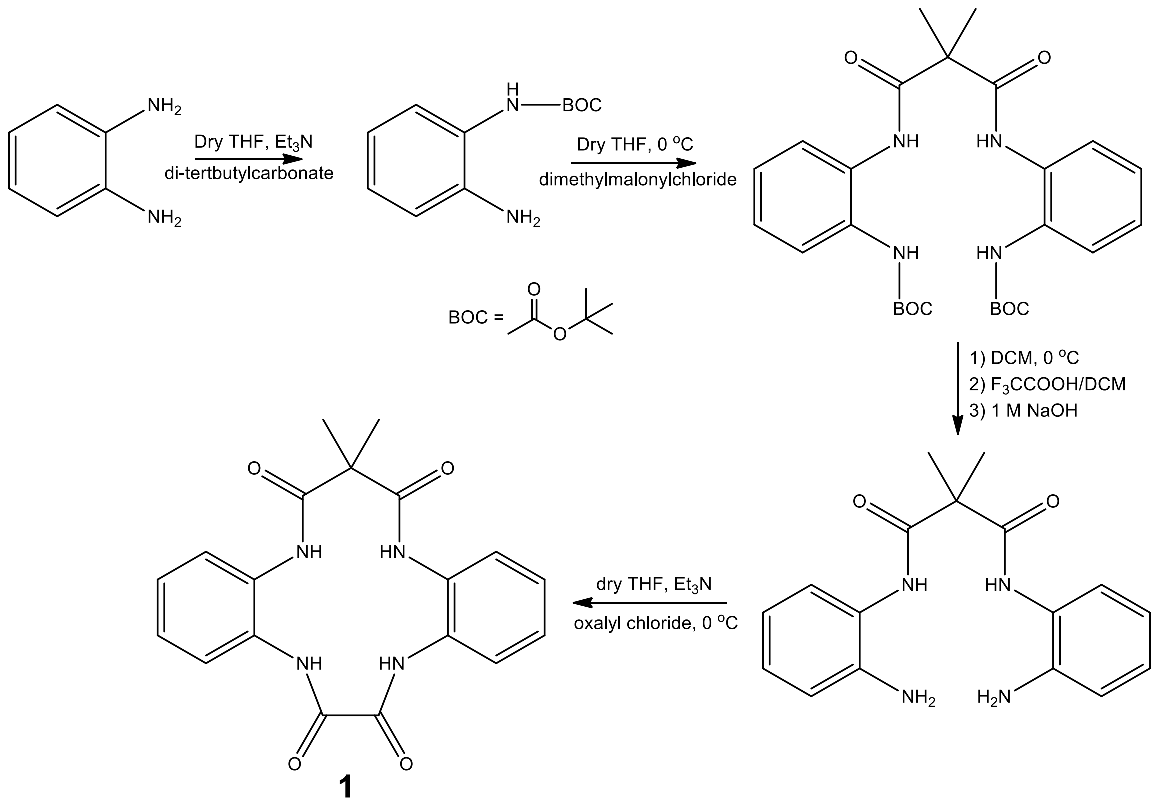

1

Figure S1. Schematic of amidomacrocyclic ligand (**1**) synthesis.[S1-S3]

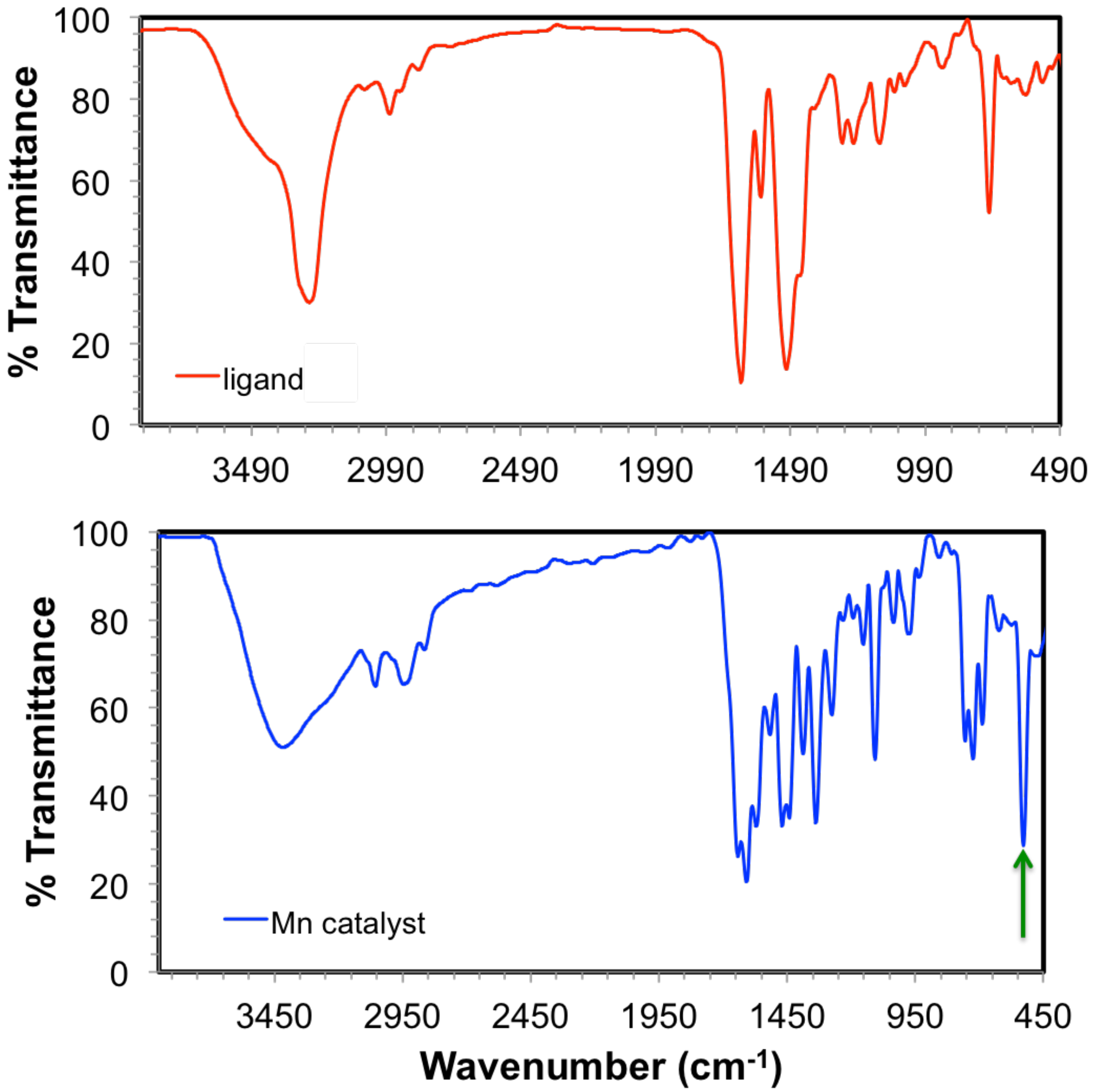

Figure S2. Infrared spectra of ligand **1** (top) and Mn catalyst **2** (bottom).

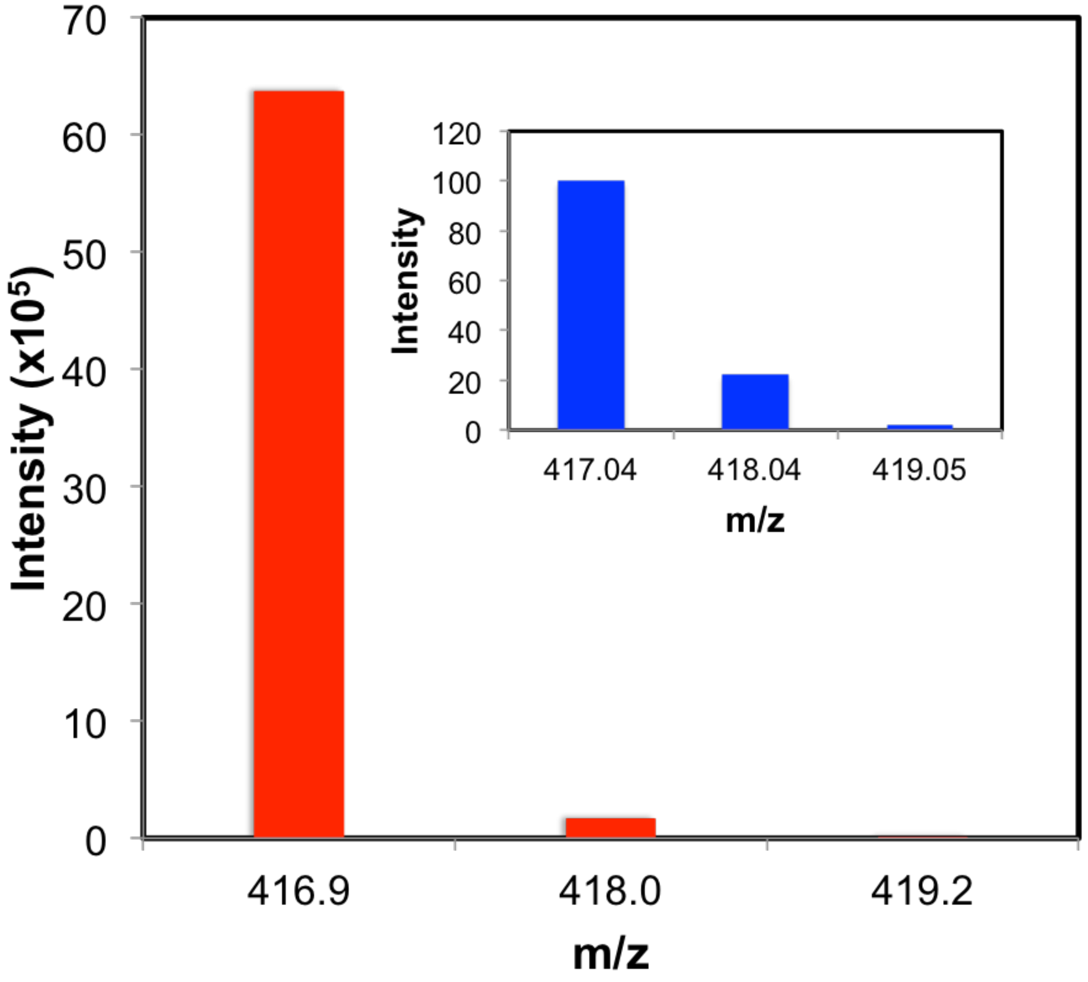

Figure S3. ESI-MS of **2**. Insert shows theoretical isotope distribution.

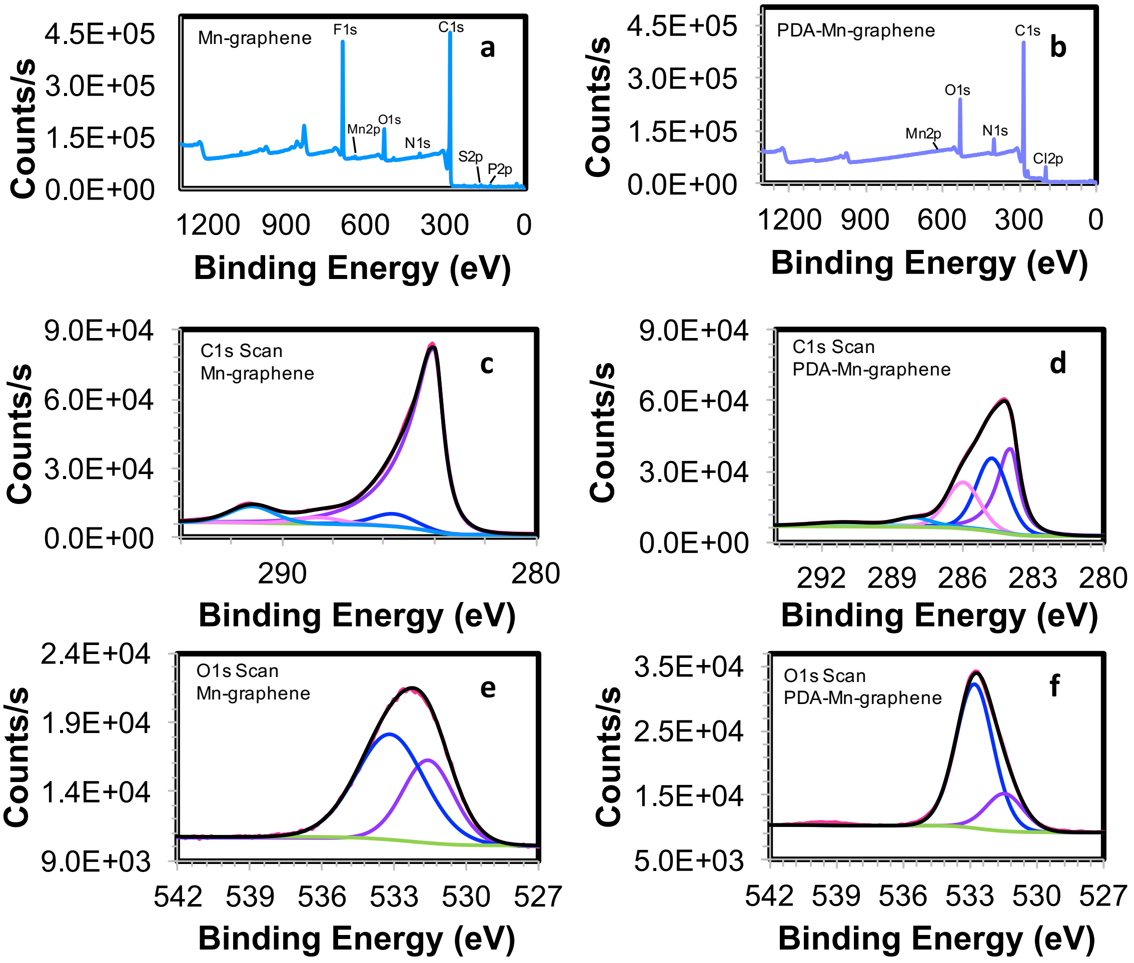

Figure S4. XPS survey scans of (a) Mn-graphene and (b) PDA-Mn-graphene nanocomposites. Narrows of Mn-graphene (c) C1s and (e) O1s and PDA-Mn-graphene (d) C1s and (f) O1s.

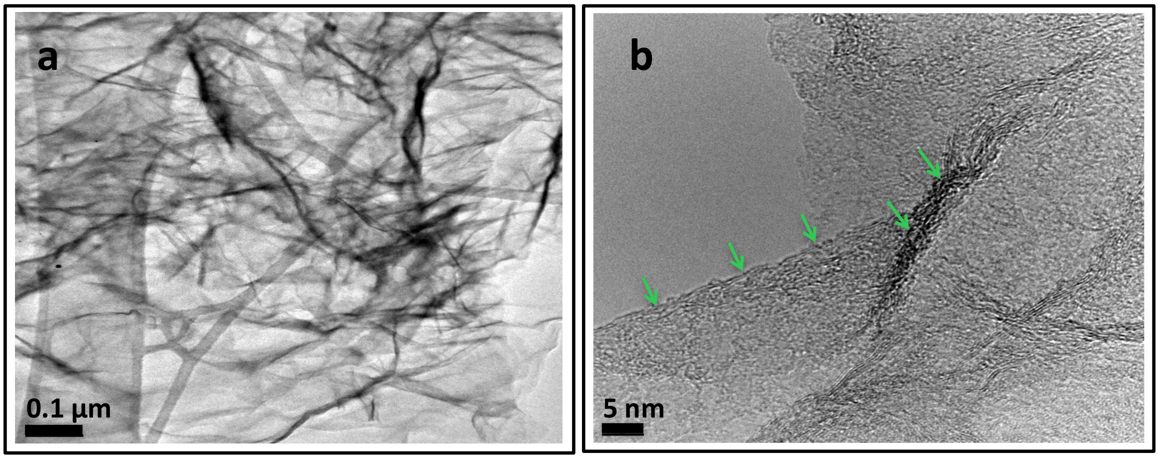

Figure S5. TEM images of (a) Mn-graphene and (b) PDA-Mn-graphene nanocomposites.

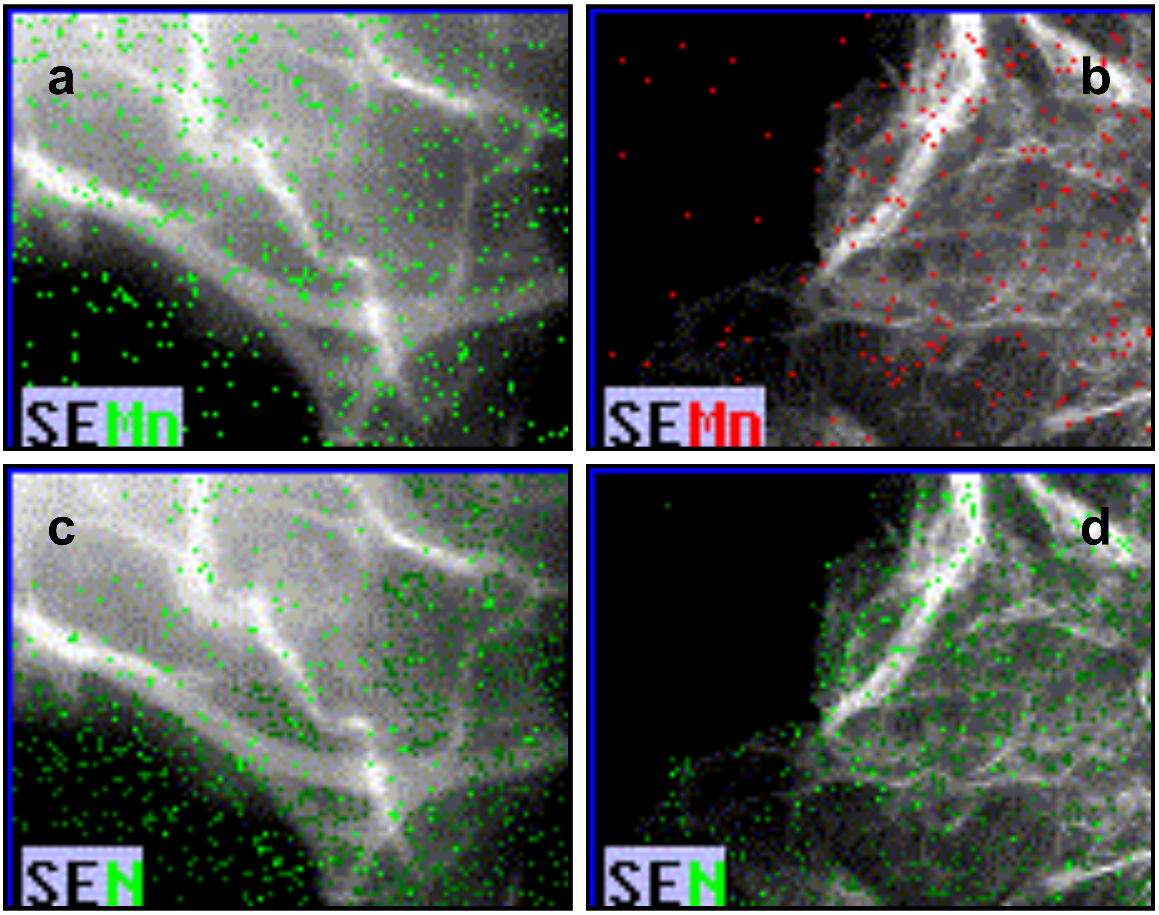

Figure S6. STEM images with EDS elemental mapping of (a) Mn and (c) nitrogen atoms in Mn-graphene and (b) Mn and (d) nitrogen atoms in PDA-Mn-graphene composite.

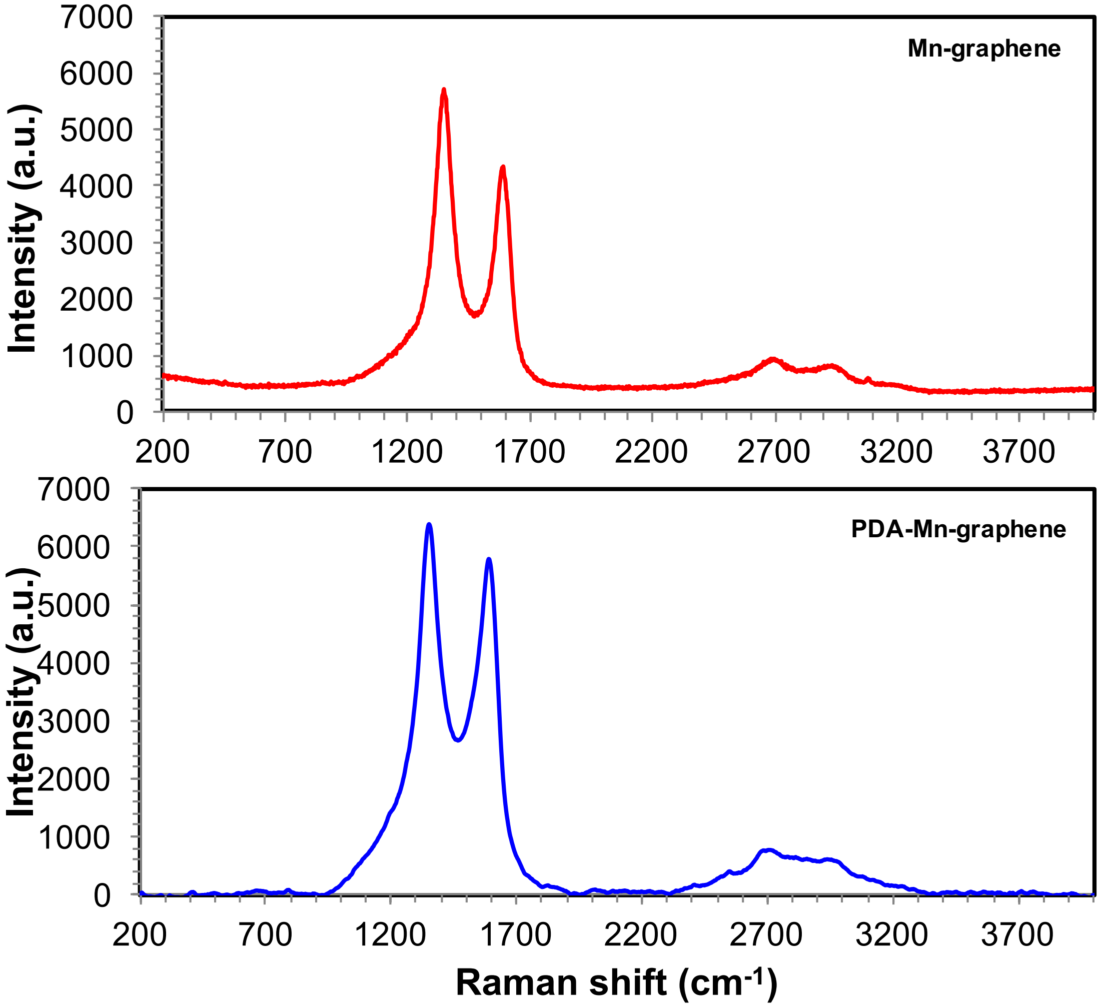

Figure S7. Raman spectra (full range) of Mn-graphene (top) and PDA-Mn-graphene nanocomposites (bottom).

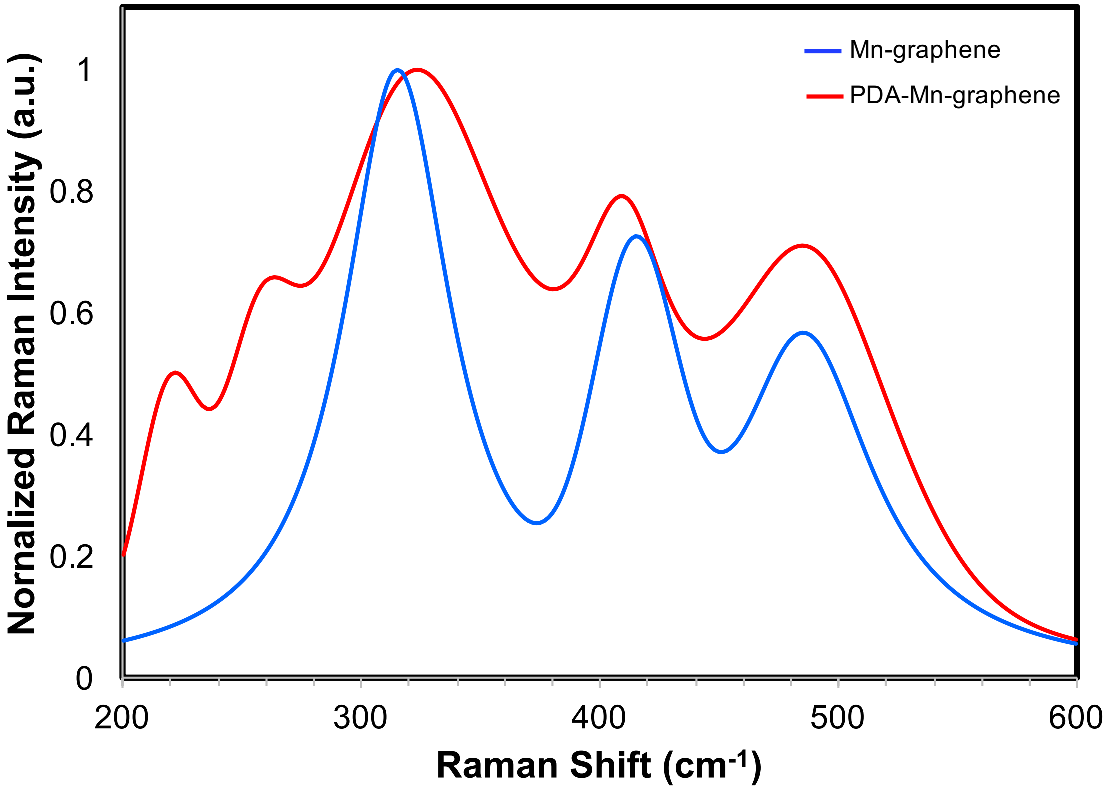

Figure S8. Raman spectra (lower range) of Mn-graphene and PDA-Mn-graphene nanocomposites.

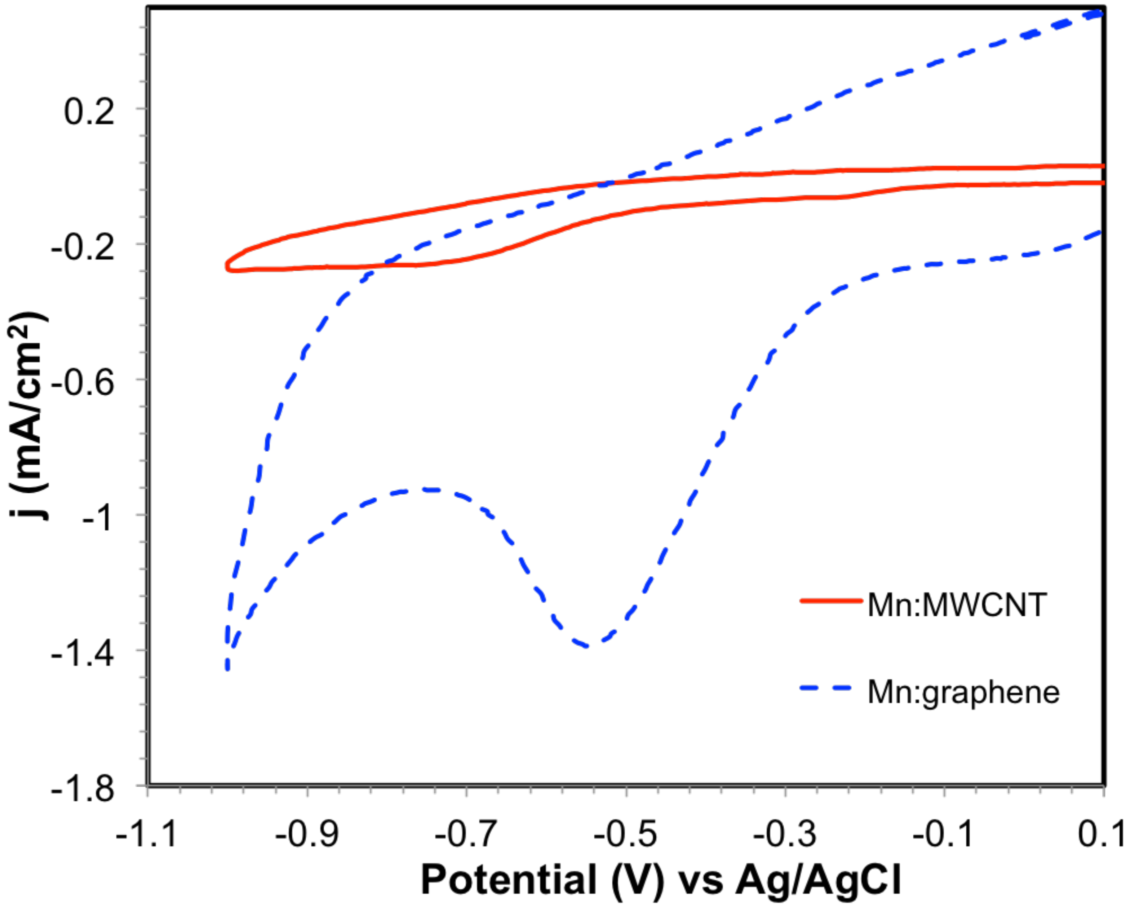

Figure S9. The effect of the carbon nanomaterial supported on **2** towards ORR.

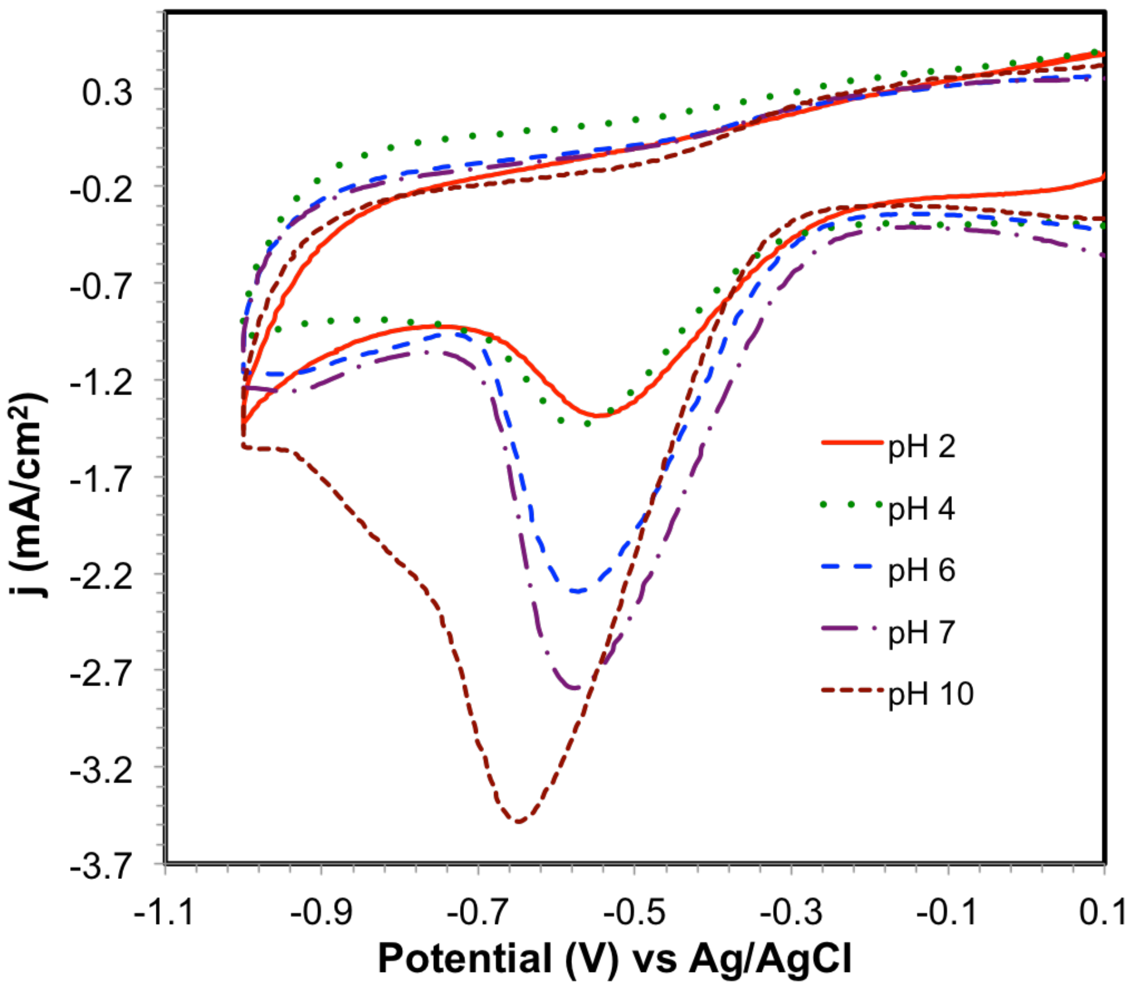

Figure S10. Effect of pH on the Mn-graphene nanocomposite.

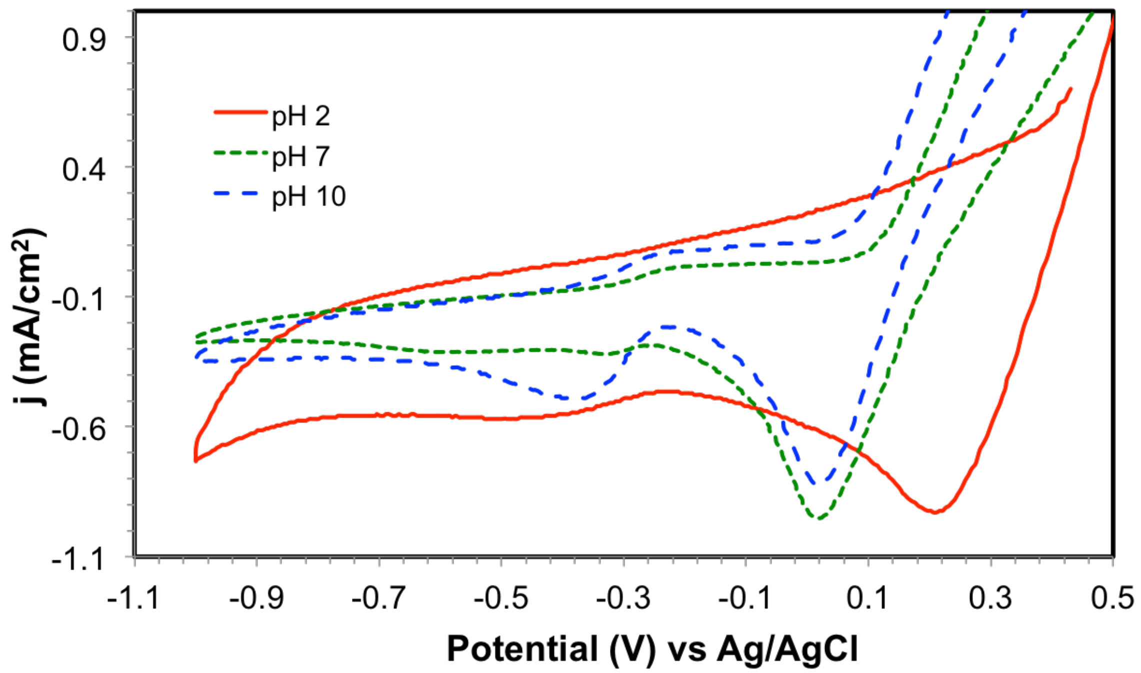

Figure S11. pH studies of PDA-Mn-graphene nanocomposite.

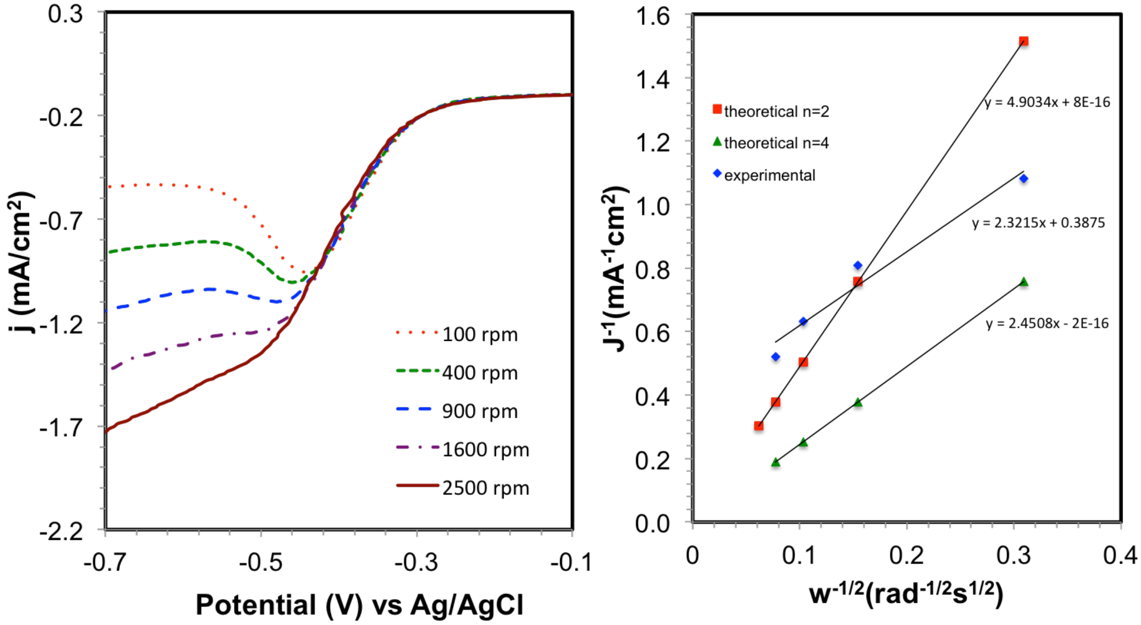

Figure S12. a) RDE studies at pH 10.0 and b) the corresponding Koutecky-Levich plot for the Mn-graphene nanocomposite.

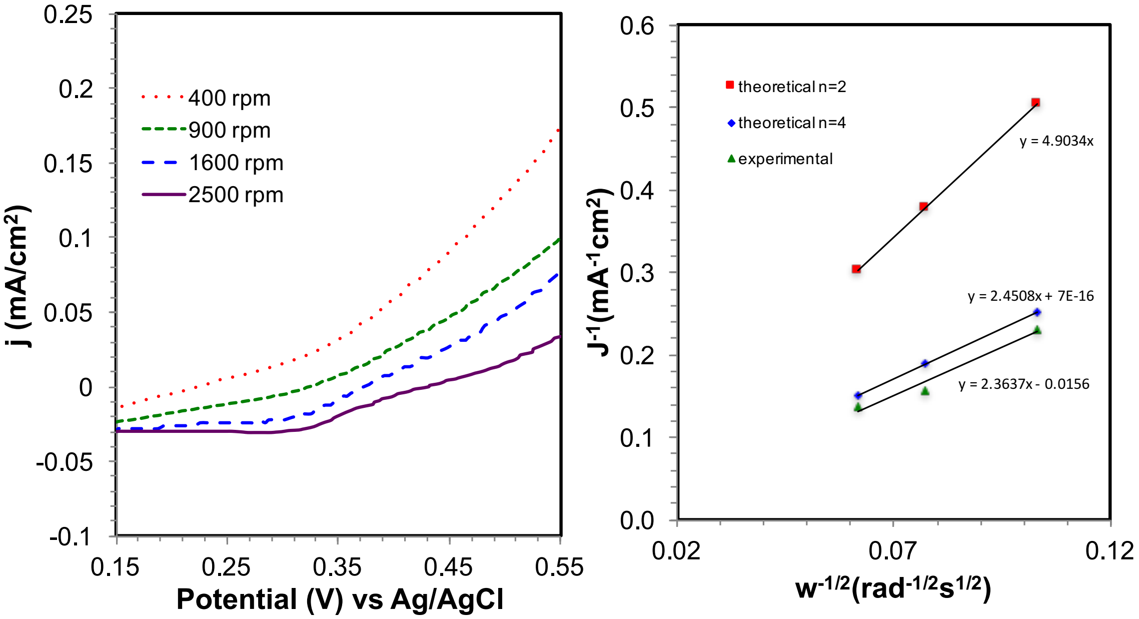

Figure S13. a) RDE studies at pH 10.0 and b) the corresponding Koutecky-Levich plot for the PDA-Mn-graphene nanocomposite.

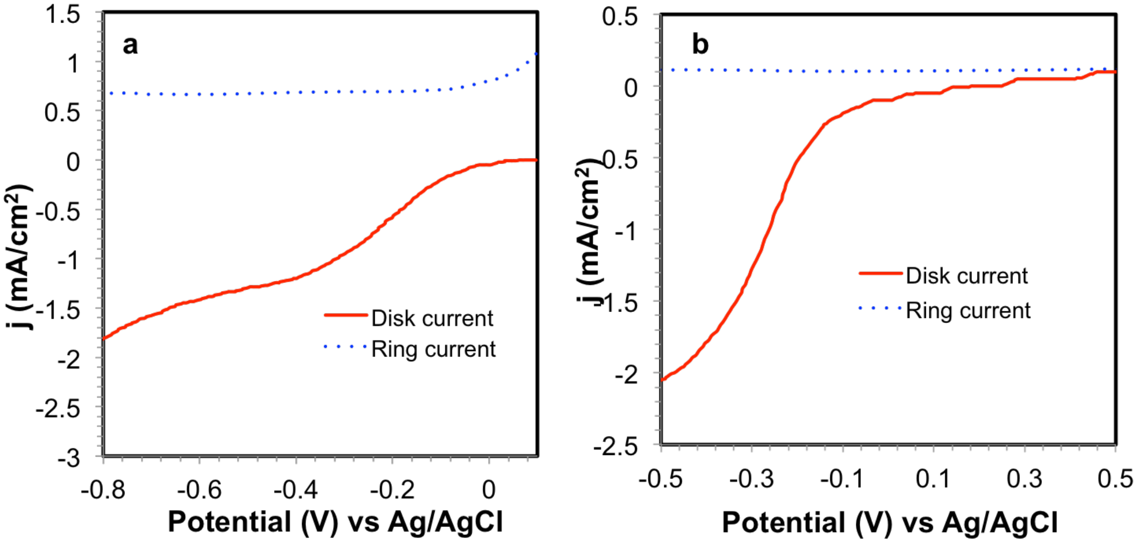

Figure S14. RRDE studies of a) Mn-graphene nanocomposite and b) PDA-Mn-graphene nanocomposite at 50 mV/s at pH 10.0.

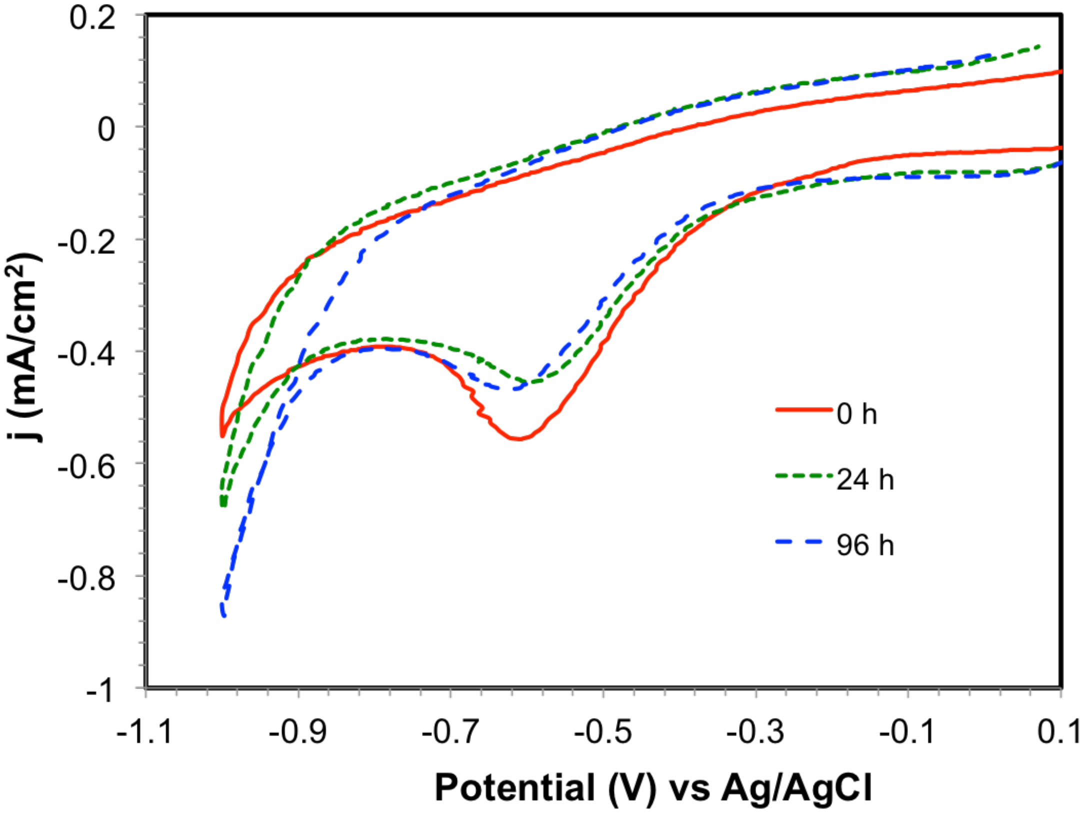

Figure S15. Stability of Mn-graphene nanocomposite in pH 2.0 after 96 h.

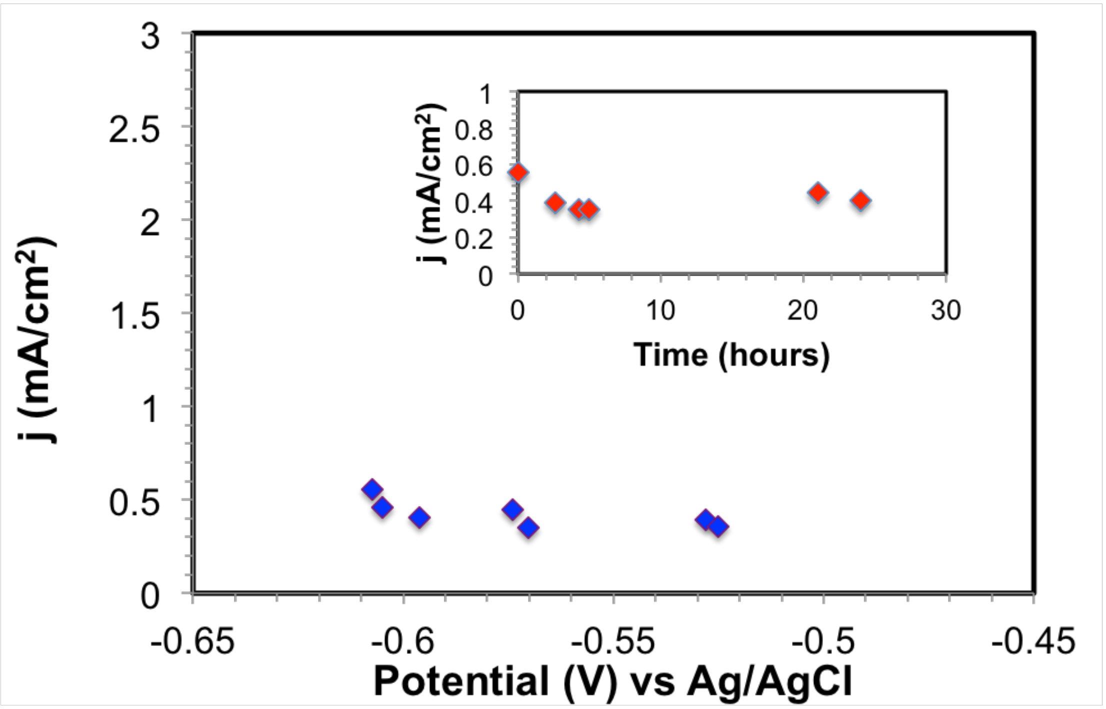

Figure S16. Changes in current density and peak potential over time of Mn-graphene nanocomposite in pH 2.0 buffer solution.

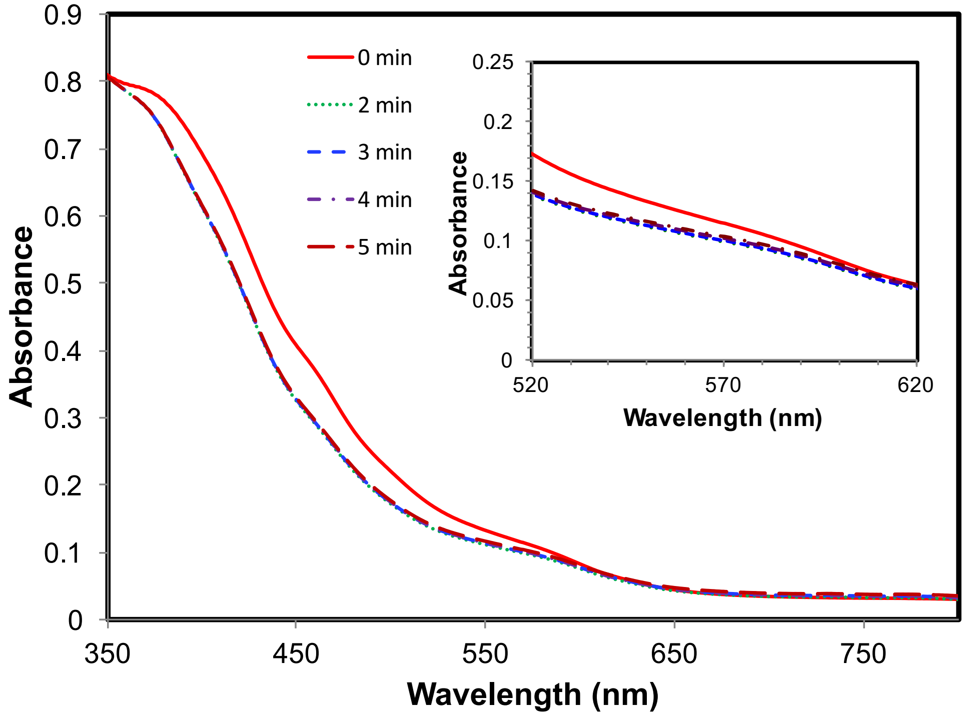

Figure S17. UV-Vis studies on Mn-graphene nancomposite stability in pH 10.0 buffer. Inset of range 520-620 nm to show no change in absorbance during kinetic studies.

Table S1. Elemental identification and quantification from XPS analysis of Mn-graphene nanocomposite.

Element	Binding Energy Peak (eV)	Atomic %
C1s	284.21	47.26
C1s Scan A	285.60	2.46
C1s Scan B	288.57	1.33
C1s Scan C	291.19	3.76
$Mn2p_{3/2}$	640.87	0.06
$Mn2p_{1/2}$	652.40	0.06
$Mn2p_{3/2}$ Scan A	642.41	0.07
$Mn2p_{1/2}$ Scan A	654.21	0.07
Mn3s	83.57	0.12
Mn3s Scan A	89.10	0.09
N1s	398.02	0.32
N1s Scan A	399.69	0.54
O1s	531.55	1.60
O1s Scan A	533.13	2.68

Table S2. Elemental identification and quantification from XPS analysis of PDA-Mn-graphene nanocomposite.

Element	Binding Energy Peak (eV)	Atomic %
C1s	284.00	27.39
C1s Scan A	284.80	27.82
C1s Scan B	286.03	18.59
C1s Scan C	288.15	3.31
C1s Scan D	291.04	2.73
Mn2p	641.47	2.29
Mn3s	83.33	1.75
Mn3s Scan A	89.13	1.74
N1s	400.03	4.56
N1s Scan A	401.73	2.21
O1s	532.81	10.45
O1s Scan A	531.46	2.73

References:

S1. Sullivan, S. Z. *et al*. Fe-complex of a tetraamido macrocyclic ligand: spectroscopic characterization and catalytic oxidation studies. *Chem. Phys. Lett.* **498**, 359-365 (2010).

S2. Ghosh, A., Sullivan, S. Z., Collom, S. L. & Pulla, S. Method of synthesis of tetradentate amide macrocycle ligand and its iron(III) complex and use as oxidation or bleaching catalyst with hydrogen peroxide. *U.S. Pat.* US 8722881 B2 (2014).

S3. Ellis, W. C.; Tran, C. T.; Denardo, M. A.; Fischer, A.; Ryabov, A. D. & Collins, T. J. Design of more powerful iron-TAML peroxidase enzyme mimics. *J. Am. Chem. Soc.* **131**, 18052-18053 (2009).
